# COVID-19 seroprevalence cohort survey among health care workers and their household members in Kinshasa, DR Congo, 2020–2022

**DOI:** 10.1186/s41043-024-00536-0

**Published:** 2024-06-01

**Authors:** Joule Madinga, Placide Mbala-Kingebeni, Antoine Nkuba-Ndaye, Leonel Baketana-Kinzonzi, Elysé Matungulu-Biyala, Patrick Mutombo-Lupola, Caroline-Aurore Seghers, Tom Smekens, Kevin K. Ariën, Wim Van Damme, Andreas Kalk, Martine Peeters, Steve Ahuka-Mundeke, Jean-Jacques Muyembe-Tamfum, Veerle Vanlerberghe

**Affiliations:** 1 Department of Epidemiology and Global Health, Institut National de Recherche Biomédicale, Faculty of Medicine, University of Kikwit, Kinshasa, Democratic Republic of Congo; 2 Department of Epidemiology and Global Health, Institut National de Recherche Biomédicale & Faculty of Medicine, University of Kinshasa, Kinshasa, Democratic Republic of Congo; 3 Virology Unit, Institut National de Recherche Biomédicale, Kinshasa, Democratic Republic of Congo; 4 Department of Epidemiology and Global Health, Institut National de Recherche Biomédicale, Kinshasa, Democratic Republic of Congo; 5 Public Health Department, Institute of Tropical Medicine, Antwerp, Belgium; 6 Virology Unit, Institute of Tropical Medicine & Department of Biomedical Sciences, University of Antwerp, Antwerp, Belgium; 7 Deutsche Gesellschaft für Internationale Zusammenarbeit (GIZ), Bonn, Germany; 8 Unit Trans VIHMI, University of Montpellier, IRD/INSERM, Montpellier, France; 9 Virology Unit, Institut National de Recherche Biomédicale & Faculty of Medicine, University of Kinshasa, Kinshasa, Democratic Republic of Congo; 10 Emerging Infectious Diseases Unit, Public Health Department, Institute of Tropical Medicine, Antwerp, Belgium

**Keywords:** COVID-19, Democratic republic of congo, Sero-survey, Cohort, Health care worker

## Abstract

**Introduction:**

Serological surveys offer the most direct measurement to define the immunity status for numerous infectious diseases, such as COVID-19, and can provide valuable insights into understanding transmission patterns. This study describes seroprevalence changes over time in the context of the Democratic Republic of Congo, where COVID-19 case presentation was apparently largely oligo- or asymptomatic, and vaccination coverage remained extremely low.

**Methods:**

A cohort of 635 health care workers (HCW) from 5 health zones of Kinshasa and 670 of their household members was interviewed and sampled in 6 rounds between July 2020 and January 2022. At each round, information on risk exposure and a blood sample were collected. Serology was defined as positive when binding antibodies against SARS-CoV-2 spike and nucleocapsid proteins were simultaneously present.

**Results:**

The SARS-CoV-2 antibody seroprevalence was high at baseline, 17.3% (95% CI 14.4–20.6) and 7.8% (95% CI 5.5–10.8) for HCW and household members, respectively, and fluctuated over time, between 9% and 62.1%. Seropositivity was heterogeneously distributed over the health zones (*p* < 0.001), ranging from 12.5% (95% CI 6.6–20.8) in N’djili to 33.7% (95% CI 24.6–43.8) in Bandalungwa at baseline for HCW. Seropositivity was associated with increasing rounds adjusted Odds Ratio (aOR) 1.75 (95% CI 1.66–1.85), with increasing age aOR 1.11 (95% CI 1.02–1.20), being a female aOR 1.35 (95% CI 1.10–1.66) and being a HCW aOR 2.38 (95% CI 1.80–3.14). There was no evidence that HCW brought the COVID-19 infection back home, with an aOR of 0.64 (95% CI 0.46–0.91) of seropositivity risk among household members in subsequent surveys. There was seroreversion and seroconversion over time, and HCW had a lower risk of seroreverting than household members (aOR 0.60 (95% CI 0.42–0.86)).

**Conclusion:**

SARS-CoV-2 IgG antibody levels were high and dynamic over time in this African setting with low clinical case rates. The absence of association with health profession or general risk behaviors and with HCW positivity in subsequent rounds in HH members, shows the importance of the time-dependent, and not work-related, force of infection. Cohort seroprevalence estimates in a ‘new disease’ epidemic seem insufficient to guide policy makers for defining control strategies.

## Introduction

Coronavirus disease (COVID-19) is an infectious disease caused by the SARS-CoV-2 virus and transmitted by respiratory droplets and aerosols [1]. Health care workers (HCWs) are among the high-risk groups for SARS-CoV-2 infection, as they are directly and/or indirectly exposed to COVID-19 patients in their working environment [2]. In sub-Saharan Africa (SSA), the COVID-19 pandemic was characterized by underreporting due to the limited testing capacity in many countries and by a large number of asymptomatic or mild symptomatic cases that are not seeking health care [3]. Hence, a significant number of patients who visit health facilities for other health problems and who could have a simultaneous asymptomatic COVID-19 infection were not tested, and they potentially exposed HCWs to the virus. On the other hand, this also holds for HCWs, who could have an asymptomatic infection and cause a risk of infection to colleagues and patients, including vulnerable individuals at high risk for severe COVID-19. Nosocomial transmission of SARS-CoV-2 accounted for 12–29% of cases in a study in China [4] and was associated with a higher mortality risk than community-acquired COVID-19 [5]. On the other hand, HCWs who acquire SARS-CoV-2 infection in health facilities bring the virus back to the communities through their close contact within households. Because of this potential role played by HCWs in the transmission of SARS-CoV-2 infection within health facilities and between health facilities and the community, it was of utmost importance to assess the importance of SARS-CoV-2 infection among HCW, comparing with community members being exposed to the general risk factors of an airborne infectious disease. Several publications are available for this target group in SSA [6–8], but most of them have a cross-sectional design, which gives information on the fraction of HCWs who tested positive at a given time during the epidemic but not on seroconversion or seroreversion over time. Studying changes in seroprevalence over time in two linked cohorts, health care workers and their household members, allows gaining insight in transmission dynamics between these two related groups of interest. Elsewhere in the first months after the start of the pandemic, as in Belgium, the seroprevalence of SARS-CoV-2 IgG among HCWs was 7.7% (95% CI: 4.8–12.1%) in April 2020 and 8.2% (95% CI: 5.7–11.6%) in September 2020 [9]. Within cohorts, seroreversion of IgG has been demonstrated, which was close to 40% over a 5-month period in a study in the USA [10]. The longevity of antibody persistence is described to be dependent on the severity of clinical signs and symptoms and has been demonstrated to be lower in asymptomatic infections [11], which are the majority of cases in SSA.

The first case of COVID-19 in the Democratic Republic of the Congo (DRC) was reported on March 10, 2020, in Kinshasa, the capital city of the country. On March 24, a state of emergency, including travel bans, was declared, and on April 6, a lockdown was installed in Gombe, which is an urban neighborhood containing the initial COVID-19 hotspot. By the end of 2021, the outbreak had spread all over the country, affecting 23 of 26 provinces and reaching a total of 79,273 confirmed cases [12], with a reported case fatality ratio of 1.5%. However, such an overall figure may hide a mortality risk of almost 50% when patients are hospitalized with severe or critical disease [13]. In November 2020, a sero-survey (Luminex assay) among the general population in Kinshasa showed a seroprevalence of 16.6% (95% CI 14.0-19.5) [14]. A study from eastern DRC in mid-2020 demonstrated a SARS-CoV-2 seroprevalence of 41.2% among HCWs in Panzi Hospital (EuroImmun IgG ELISA), with 22.3% of seropositive HCWs reporting symptoms congruent with COVID-19 illness [8].

In this study, we aimed to determine the prevalence of SARS-CoV-2 antibody levels, together with seroconversion/seroreversion dynamics among a cohort of HCWs and their household members in Kinshasa at several time points up to almost two years after the start of the pandemic. As a secondary objective, we evaluated the risk factors for seroprevalence and seroconversion/reversion in both groups and defined the temporal relation between a SARS-CoV-2 IgG positive health care worker and the seroconversion of their household members. In the discussion, we revisit the evidence generated in this study and the usefulness of seroprevalence studies for the definition of control program strategies [15].

## Methods

### Ethical statement

The current study was approved by the ethics committee of the University of Antwerp, Belgium (number B3002020000144) and the national DRC ethics committee (189/CNES/BN/PMMF/2020). Further approval was obtained from the Ministry of Health through the provincial health division as well as the heads of concerned health zones (HZ) and health facilities. Prior to inclusion, the purpose of the study was explained to each potential participant, and written informed consent was obtained.

### Study area

**Fig. 1:**
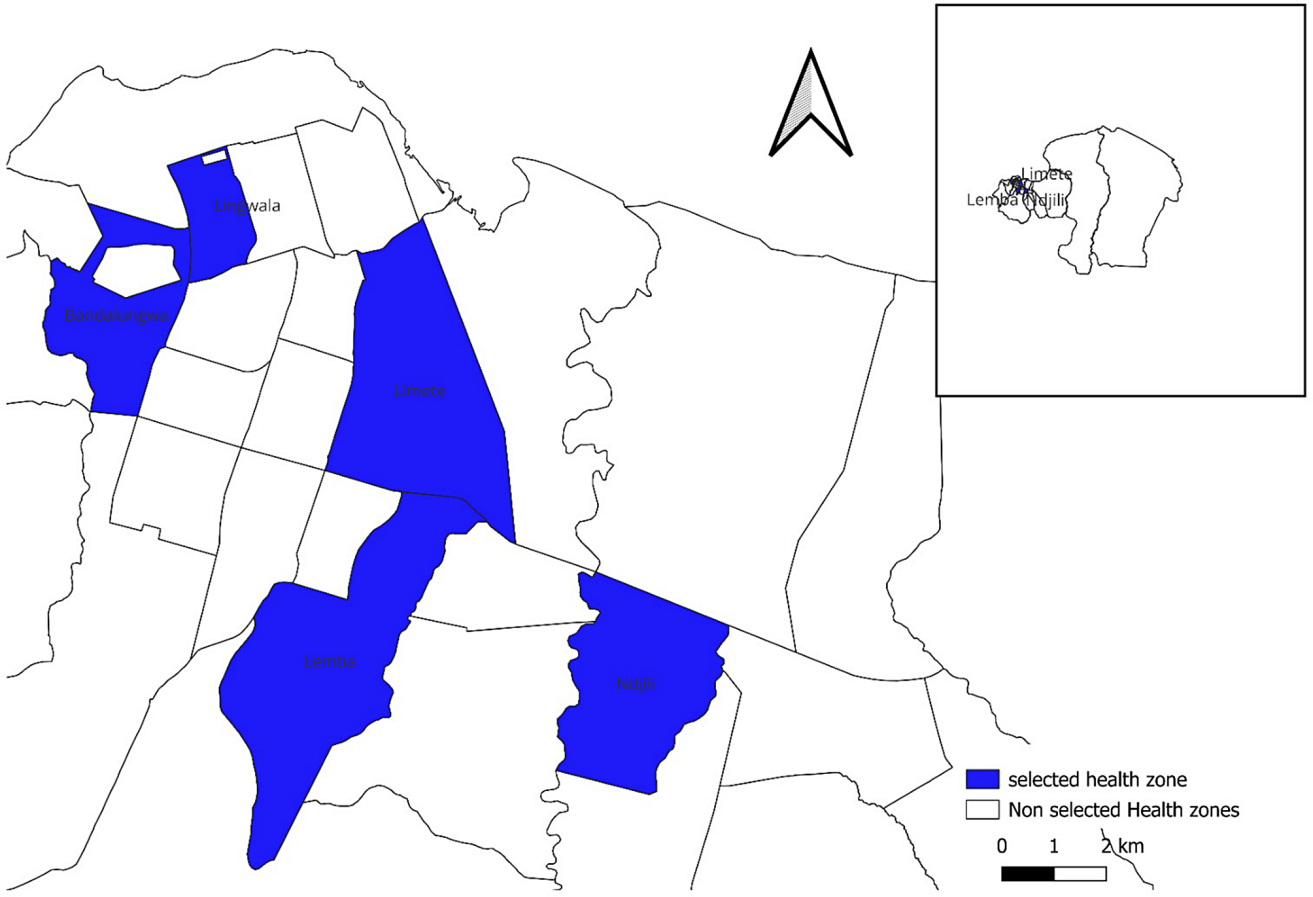
Geographical location of the five study health zones in Kinshasa, DRC, 2020–2022

The study was conducted in five health zones (HZs) of Kinshasa (Fig. 1), purposively chosen on the basis of the presence of COVID-19 treatment services in the main hospital of the HZ (N’djili, Lemba, Limete, Lingwala) and reports of COVID-19 cases in the first weeks of the pandemic (Bandalungwa). In the DRC, an HZ is the operational unit implementing primary health care strategies within the health system. HZs are divided into health areas covering a number of streets in urban areas. Each HZ comprises a central office, a general reference hospital, and at least one health center per health area. In 2020, the population of Kinshasa was estimated at 12,117,417 inhabitants by the National Health Information System and is distributed over 35 HZ. Kinshasa is a city that faces poverty, high population density, lack of an organized interurban transport network, and a low level of public hygiene standards. The city is also an important entry point through its international airport and port. In response to the COVID-19 epidemic, COVID-19 response teams were set up in each HZ of Kinshasa. They were responsible for the contact tracing of confirmed cases, investigations and detection of febrile/suspect cases in the community.

### Study design, population and data collection

**Fig. 2:**
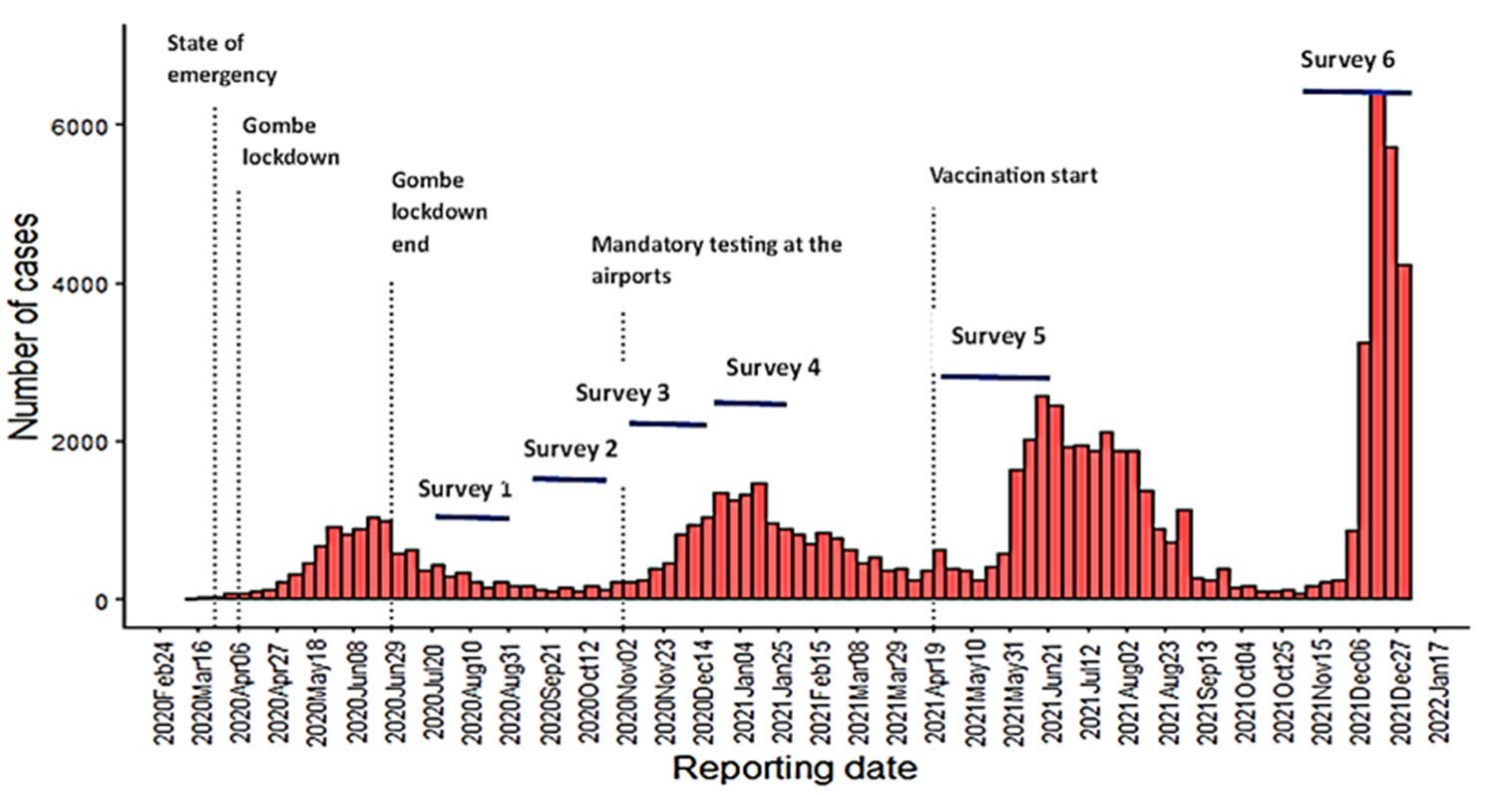
Epicurve of COVID-19 cases in DRC, reported by the Ministry of Health, with indication of the period of study surveys, 2020–2022

This study was designed as a prospective cohort study (Fig. 2). The cohort of participants was interviewed and seroprevalence through SARS-CoV-2 IgG antibody levels assessed, at 6 time points, the first 4 at 6- to 8-week intervals and the last 2 at 4- and 8-month intervals. The study population consisted of HCWs from the different structures in the five selected health zones (HZs) and their household members (HH members). In each selected HZ, one hospital, two healthcare centers (with the highest frequency of consultations), and the COVID-19 response team of the HZ were included. In total HCWs were hence selected among 5 hospitals, 10 health care centers and 5 COVID-19 response teams. An HCW was defined as any category of staff working in a health establishment, whether or not they were in direct contact with patients and their objects. A household member was defined as a member of the health care worker’s household, which consists of groups of individuals who live together in the same house, share the same housekeeping arrangements and usually eat meals together. The sample size for our baseline survey in HCW and HHmembers was based on estimating an expected seroprevalence of 5% in the HCWs (estimated to be higher than the seroprevalence for HHmembers) with a precision of 2% (alpha error of 0.05) and a design effect of 1.4, leading to a minimum sample of 650 participants in both groups. For the cohort, for an expected difference of 1% seroconversion in HH members and 4% seroconversion in HCWs (both starting at an expected seroprevalence of 5%) in consequent surveys, to detect this with a power of 80% and 95% precision, 339 HCWs and 678 HH members were needed, and considering the 20% probability of loss to follow-up, the total study sample estimated was 650 HCWs and 975 household members. This sample size is also big enough to detect risk factors in each survey round with a presence of 50% and precision of 5%, using the same alpha error and power as stated above.

Within each facility, HCWs were randomly selected based on the payroll list stratified by department. A total of at least 118 HCW per HZ were selected as following: In the hospitals, at least 12 participants were randomly selected in each of 8 wards; in the health centers, at least 6 participants were selected in the consultations and 6 in the laboratory/reception/social service; in the COVID-19 response team, at least 10 participants were randomly selected. For the household members of the HCWs, a subsample was selected in order to reach the required sample size: 2 HCWs per ward in each health facility were randomly selected (ad random function Excel), and for those selected HCWs, all their household members were invited to come to the health infrastructure for the survey. The cohort of participants was followed up at 6 time points, the first 4 at 6- to 8-week intervals and the last 2 at 4- and 8-month intervals. At each survey or round, all participants answered the questionnaire, and a blood sample was taken and tested. The questionnaire for HCWs was adapted from the WHO surveillance protocol for SARS-CoV-2 infection among health workers [16, 17] and for HH members from the WHO household transmission investigation protocol [18]. In addition to demographic and medical history characteristics, the questions were probing for work-related exposure in relation to COVID-19 patients and the concomitant risk factors for HCWs and for general COVID-19 transmission risk behavior for HH members. Participants’ reports on typical symptoms of COVID-19 infection and COVID-19 PCR testing over the month prior to the survey were also included in the survey.

The study period was from July 2020 to January 2022.

### Laboratory analysis

Upon informed consent, a 6 ml whole blood sample was taken from each HCW and a finger prick sample (minimum 3 - max 6 whole blood spots (75 µl)) for HH members (minimal discomfort and higher acceptability for non-health professionals) by an experienced nurse or laboratory technician. The collected blood specimens and dried blood spots were stored in the health facility laboratory in temperature-controlled (4 °C) conditions before daily transportation to the reference ‘Institut National de Recherche Biomédicale’ (INRB) laboratory. Filter papers were stored as aliquots of serum samples after centrifugation in a -20 °C freezer until further analysis.

For analysis, blood spot filter papers were prepared by punching two discs of 4-mm diameter and eluted overnight in 160 µL of PBS-TBN (dilution 1:40, PBS-1% BSA-0.15% Tween, pH 7.4, Sigma‒Aldrich). Just before use in the immunoassay, the eluted samples were further diluted to a final plasma dilution of 1:200 in PBS-BN, similar to the serum samples. The presence of binding antibodies to SARS-CoV-2 was tested with a highly sensitive and specific in-house Luminex multiplex antibody-based assay used to simultaneously detect IgG antibodies to two viral antigens, i.e., recombinant Nucleocapsid (NP) and Spike (SP) proteins derived from SARS-CoV-2 (see detailed information in publications [14, 19]). A sample was considered positive for immunoglobulin G (IgG) against SARS-CoV-2 if it reacted simultaneously with NP and SP proteins and negative if it reacted to only one protein or if the median immunofluorescence intensity was below the cutoff for both antigens.

### Data analysis

A descriptive analysis of the demographic and exposure/risk factors for both the HCW and HH groups was performed stratified by seropositivity at baseline. Various composite indicators were made. The variable ‘Contact with patients in health facility’ was defined as [1] direct contact (professional groups who are in close contact with patients, namely, medical doctors, nurses and assistant nurses) [2], indirect contact (professional groups of laboratory and hygiene personnel) and [3] no or little contact (including professional groups of maintenance, administration, and community workers). The variable ‘Ward risk related to COVID-19’ was defined as ‘low’ if health personal was working in administration, pharmacy, radiology and cleaning services, as ‘medium’ in outpatient department, laboratory, or surgical wards, as ‘high’ in COVID wards, intensive care units and emergencies. The variable ‘Personal Infection control material availability’ is based on 9 questions about availability of water, soap, disinfectant, masks, gloves, face screens together with available training and knowledge on standard protection measures; ‘minimal’ was defined as answering positive on less than 4 questions, ‘good’ for 5–7 questions, and ‘very good’ for answering positive on 8 or 9 questions. The variable ‘Personal Protection practice’ ’ was based on 7 exposure questions to patient and/or their belongings and/or patient body fluids together with direct contact to COVID-patients (regrouped as: value of ‘basic’ if no use and exposure or not to COVID, and ‘good’ if PPE use with clear exposure to COVID). Probing for risk exposure to COVID-19 patients, a composite indicator ‘Exposure score to COVID-19 patients’ was made based on the presence of a COVID-19 ward in the hospital, being affected or not to such a ward, use of personal protection material, and realizing invasive procedures or not. A score of 4 or less was categorized as low exposure, and a score of 4 or more was categorized as high exposure to COVID-19. For HH members, ‘risk behavior’ was defined as having at least a score of 3 on the composite indicator, calculated by the addition of 6 risk behavioral variables: working/being outside the house for work/travel/studying, not washing hands, not wearing masks in the street, not wearing masks on public transport, participation in public gatherings, and not keeping a 1.5 m distance when outside the house. The variable ‘History of COVID-19 like symptoms in last month’ is based on the WHO case definition, namely, having had at least 3 of the COVID-19 symptoms (fever, cough, fatigue, headache, muscle pain, sore throat, runny nose, shortness of breath, vomiting/nausea, diarrhea and/or alteration of consciousness) in the last 4 weeks.

Subsequently, seroprevalence was calculated for the total sample and each group at each survey. Confidence intervals were obtained by the Clopper-Pearson procedure, and *p* values were obtained by the chi-square test or an F test. A multilevel logistic regression model was used to estimate determinants for seroprevalence status, with random effects at the participant level. Only independent variables collected in both groups were tested (see Table 1). The R package lme4 was used [20].

**Table 1:** Baseline seroprevalence of SARS-CoV-2 infection by demographic and work profile characteristics of health care workers and risk behavior profile of household members

Health care workers				
Factors	n	Positive	% (95%CI)	P**
**Sexe**				
Male	241	40	16.6 (12.1–21.9)	0.736
Female	320	57	17.8 (14-22.4)	
**Age group**				
18–30	74	8	10.8 (4.8–20.2)	0.061
31–40	180	30	16.7 (11.5–22.9)	
41–50	135	20	14.8 (9.3–21.9)	
51–60	99	19	19.2 (12-28.3)	
> 60	62	18	29.0 (18.2–41.9)	
**Working health zone**				
Bandalungwa	101	34	33.7 (24.6–43.8)	**< 0.001**
Lemba	138	19	13.8 (8.5–20.7)	
Limete	96	12	12.5 (6.6–20.8)	
Lingwala	126	20	15.9 (10-23.4)	
Ndjili	100	12	12.0 (6.4–20.0)	
**Working health facility**				
Hospital	302	52	17.2 (13.4–21.9)	0.850
Health center	199	36	18.1 (13.0-24.1)	
COVID-19 response team	60	9	15.0 (7.1–26.6)	
**Contact with patients in health facility**				
Direct contact	308	49	15.9 (12.2–20.4)	0.522
Indirect contact	83	14	16.9 (9.5–26.7)	
No contact	170	34	20.0 (14.3–26.9)	
**Ward risk related to COVID-19**				
Low risk	174	34	19.5 (13.9–26.2)	0.481
Medium risk	254	44	17.3 (12.9–22.5)	
High risk	133	19	14.3 (8.8–21.4)	
**Personal infection control material availability**				
Minimal	38	6	30.8 (9.0–61.4)	0.900
Good	285	52	18.2 (13.9–23.1)	
Very good	133	19	14.3 (8.8–21.4)	
**Personal protection practice**				
Basic	13	4	30.8 (9.0–61.4)	0.252
Good	374	63	16.8 (13.4–21)	
**Exposure score to COVID-19 patients**				
Low exposure	351	66	18.8 (15.0–23.2)	0.249
High exposure	210	31	14.8 (10.3–20.3)	
**History of COVID-19 like symptoms in last month**				
Yes	57	11	19.3 (10.0–31.9)	0.711
No	504	86	17.1 (14.0–20.6)	
**Householdmembers**				
**Sexe**				
Male	192	10	5.2 (2.5–9.4)	0.071
Female	233	24	10.3 (6.7–14.9)	
**Age group**				
< 18	208	17	8.2 (4.8–12.8)	
18–30	117	11	9.4 (4.8–16.2)	0.711
31–40	47	3	6.4 (1.3–17.5)	
41–50	20	0	0.0 (0.0-16.8)	
51–60	10	2	20.0 (2.5–55.6)	
> 60	5	0	0.0 (0.0-52.2)	
Risk behavior*				
No or few risk behavior	345	29	8.4 (5.7–11.8)	0.651
Risk behavior	80	5	6.2 (2.1–14.0)	
**History of COVID-19 like symptoms in last month**				
Yes	7	0	0.0 (0.0–41.0)	0.988
No	418	34	8.1 (5.7–11.2)	

*risk behavior: 3 or more risk behaviors (working/being outside the house for work/travel/studying, not washing hands, not wearing masks in the street, not wearing masks on public transport, participation in public gatherings, and not keeping a 1.5 m distance when outside the house)

**p-value of bivariate analysis

To evaluate whether there was transmission from HCWs to their families, a subgroup analysis was performed, adding the variable ‘COVID seroprevalence result of HCWs in the previous round’ to the abovementioned multilevel logistic regression model. The inclusion criteria for this subgroup were being household members, who are themselves nonhealthcare workers, and for whom there was a result of sero-survey of the HCW (of this household) in the previous round.

To visualize the seroreversion and seroconversion patterns, an alluvial plot was constructed.

To evaluate the determinants of seroreversion, seroreversion was defined as ‘a participant’s current test result is negative, and their previous test result is positive (allowed to “jump over” previous rounds with no available test result)’. Every round where both the current and a previous test result were available and where the previous test result was positive is therefore an opportunity to have observed a seroreversion. The number of such rounds was considered the “population size” for that participant. The seroreversion rate, participants’ number of seroreversions, was modeled using Poisson regression, while including the log of “population size” as the offset.

NP and SP antibody levels follow an exponential distribution, and to analyze the ratio of change over time, a zero-inflated Poisson regression model was used. To account for the longitudinal nature of the data, we included random effects in the model for participant ID (but not for health zone and household, as the model was not converging). The R package GLMMadaptive was used to perform this analysis [21].

Data analysis was conducted using R software version 4.1.1.

## Results

### Baseline participant characteristics and seroprevalence of SARS-CoV-2 infection

Baseline demographic, work profile and work-related COVID-exposure (for HCWs) and risk behavior (for HH members) characteristics in relation to SARS-CoV-2 seropositivity at baseline are shown in Table 1 (bivariate analysis). Of a total of 561 HCWs and 425 HH members, 320 (57.0%) and 233 (54.8%) were female, respectively. The ages of the HCW and HH participants ranged from 18 to 84 years with a median of 43 years (IQR = 34–50) and from 0 to 85 years with a median of 18 years (IQR = 11–28), respectively. More than half of the HCWs included (302/561) were working in a hospital, and 308 had direct contact with patients. Eighty (18.8%) of the HH members reported no or minimal protection against COVID-exposure, such as washing hands, wearing masks, and keeping a distance of 1.5 m. A total of 57 HCWs and 7 HH members reported symptoms similar to those of a COVID-19 infection during the month prior to the interview, but this was not significantly associated with seroprevalence. Overall, 97 HCW participants were positive (17.3%, 95% CI 14.4–20.6) at baseline, and the highest seroprevalence was found in the Bandalungwa health zone (*p* < 0.001). Seropositivity was lower for HH members, attaining 7.8% (95% CI 5.5–10.8) at baseline. Only 35 participants reported at baseline having been tested for acute COVID infection on the basis of a PCR test.

### Patterns of SARS-CoV-2 seroprevalence over time

**Fig. 3:**
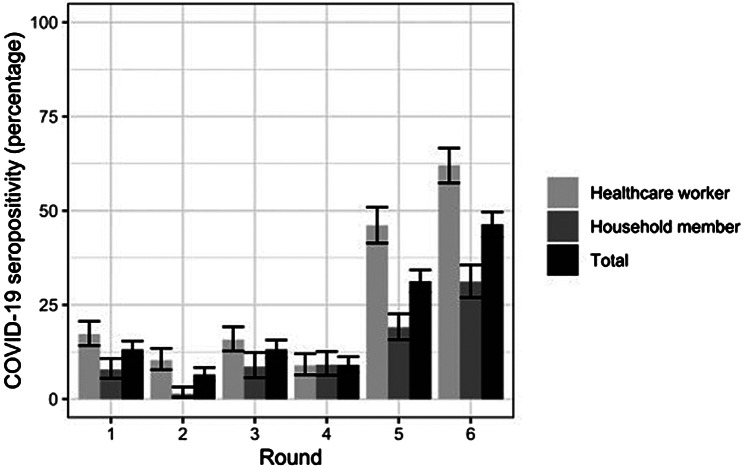
COVID-19 seroprevalence in health care workers and their household members, Kinshasa, DRC, 2020–2022. Rounds: 1 = July/August 2020 (*n* = 996); 2 = September/October 2020 (*n* = 834); 3 = November/December 2020 (*n* = 828); 4 = December 2020/January 2021 (*n* = 787); 5 = April/June 2021 (*n* = 976); 6 = November 2021/January 2022 (*n* = 902)

The seroprevalence pattern among HCWs and HH members over time had an increasing trend, reaching 62.1% and 31.2% in the last survey in health care workers and household members, respectively. Household members always had lower seroprevalence than health care workers, except in round 4, where seroprevalence was very similar in both groups (Fig. 3). Of the 902 participants in the last survey, 45 (5.0%) – 39 HCWs and 6 HH members – reported having received at least one COVID-19 vaccination.

### Determinants of seropositivity and patterns of seroconversion and seroreversion

The multivariable analysis (Table 2) shows that seropositivity increased significantly with increasing rounds, increasing age, being a female (in comparison to male) and being a health care worker (in comparison to being a HH member).

In a subanalysis on the household members to evaluate whether transmission is suggestive of coming from health care workers toward other family members, it was observed that if a health care worker was positive in a previous round, household members were not more infected. It was even the opposite when controlling for the confounding factors (same as in Table 2), resulting in a crude OR of 0.87 (95% CI 0.64–1.19) and an adjusted OR of 0.64 (95% CI 0.46–0.91), *n* = 563 participants, 1709 observations.

**Table 2:** Determinants of COVID-19 seropositivity (*n* = 1220, 5116 observations), Kinshasa, 2020–2022

Parameter	Crude OR (95% Wald CI)*	Adjusted OR (95% Wald CI)*
Round**	1.71 (1.63–1.81)	1.75 (1.66–1.85)
Age (per 10 years)	1.22 (1.16–1.29)	1.11 (1.02–1.20)
Female (vs. male)	1.26 (1.04–1.53)	1.35 (1.10–1.66)
Healthcare worker (vs. HH member)	2.16 (1.79–2.61)	2.38 (1.80–3.14)
**Random effects**		
Variance between participants	0.88	1.17

*multilevel logistic regression model

**continuous variable, from round 1 to round 6

Over the entire study period, there were 372 participants, HCWs and HH members, who had six data and sample collections. Of them, five participants (1,3%), only HCWs, stayed positive from the start to the end, and 114 (30.6%) stayed negative over the entire period.

**Fig. 4:**
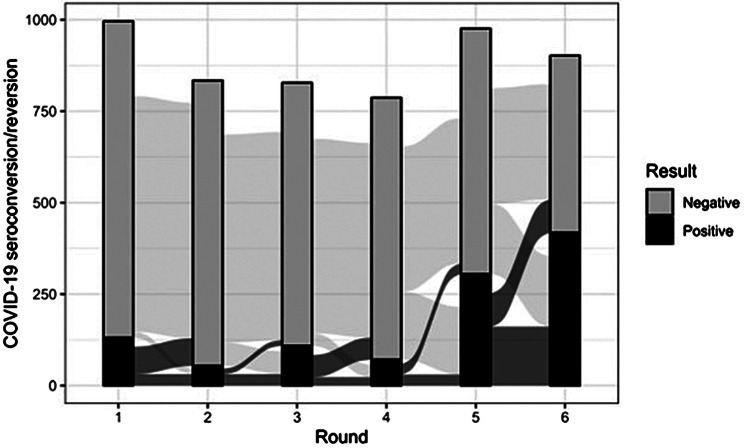
COVID-19 Sero-conversion and sero-reversion in healthcare workers and their household members, Kinshasa, DRC, 2020–2022. Rounds: 1 = July/August 2020 (*n* = 996); 2 = September/October 2020 (*n* = 834); 3 = November/December 2020 (*n* = 828); 4 = December 2020/January 2021 (*n* = 787); 5 = April/June 2021 (*n* = 976); 6 = November 2021/January 2022 (*n* = 902)

Sero-reversion was important and was observed from the second round onward (Fig. 4). In a subsample of participants (both HCWs and HH members), the association of seroreversion with demographic characteristics showed that health care workers were estimated to have a 40% lower seroreversion rate than household members. There was no evidence for an association with age or gender (Table 3). The low number of observations is because only 397 participants ever tested positive and had at least one test result in a subsequent round, meaning they had at least one opportunity to manifest a seroreversion.

**Table 3:** Determinants of COVID-19 seroreversion (*n* = 397 participants in the subanalysis), Kinshasa, 2020–2022

Parameter	Crude OR (95% Wald CI)*	Adjusted OR (95% Wald CI)*
Age (per 10 years)	0.88 (0.82–0.94)	0.97 (0.88–1.07)
Female (vs. male)	0.99 (0.77–1.28)	0.95 (0.74–1.22)
Healthcare worker (vs. HH member)	0.55 (0.43–0.71)	0.60 (0.42–0.86)

*Poisson regression

### Antibody levels over time

**Fig. 5:**
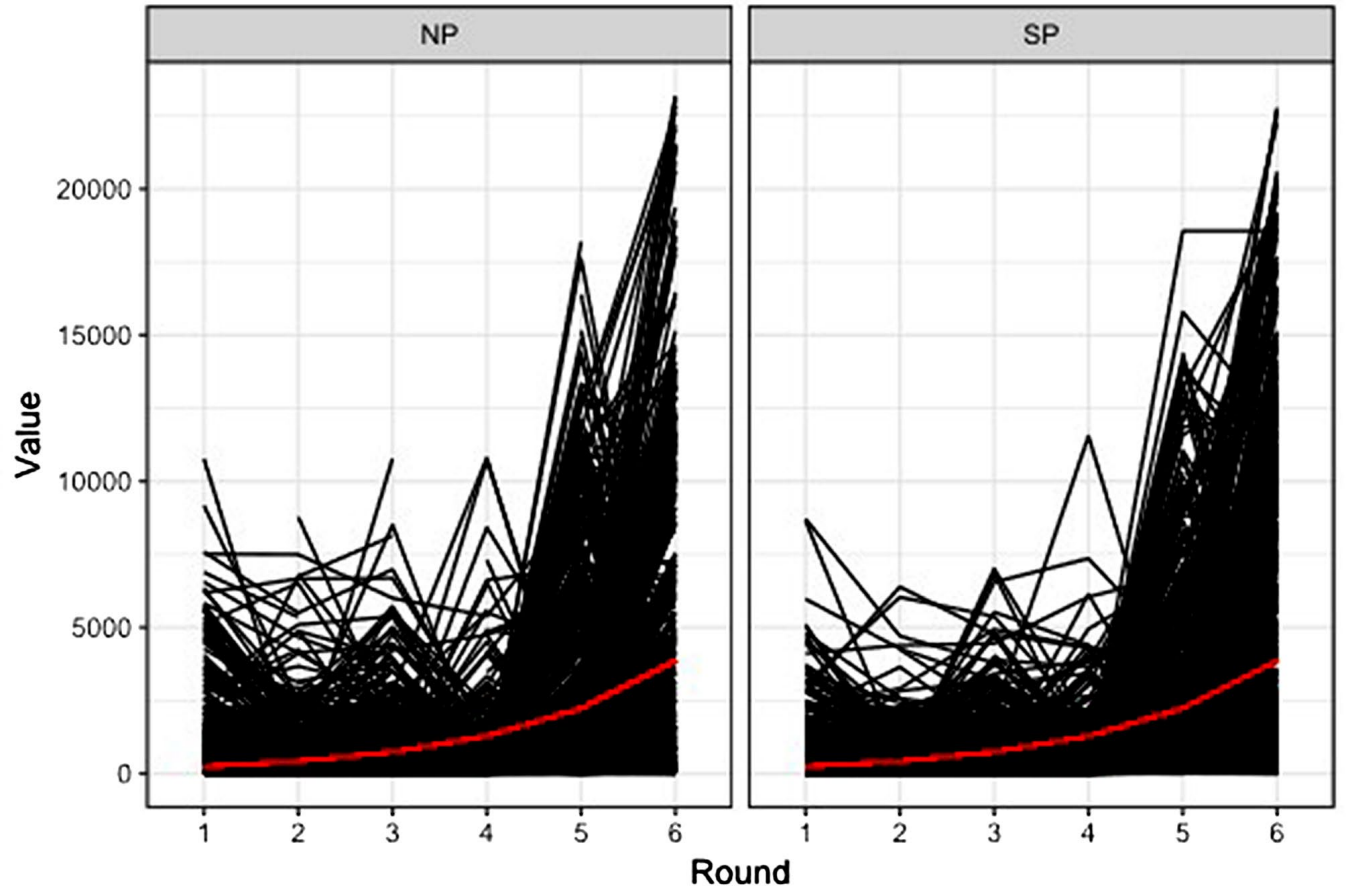
Participants’ trend in COVID-19 NP and SP antibody levels, Kinshasa, DRC, 2020–2022. Rounds: 1 = July/August 2020 (*n* = 996); 2 = September/October 2020 (*n* = 834); 3 = November/December 2020 (*n* = 828); 4 = December 2020/January 2021 (*n* = 787); 5 = April/June 2021 (*n* = 976); 6 = November 2021/January 2022 (*n* = 902)

In the multiplex serological test used, both NP and SP antibodies were evaluated. A positive COVID-test result was based on being positive (above threshold) for both antibodies. Over time, we observed an increase in antibody levels among the positive samples (Fig. 5). With each round, the odds of having NP antibodies increased by 60%, odds ratio of 1.60 (95% CI 1.33–1.93), and the level of NP antibodies increased by 53%, rate ratio of 1.53 (95% CI 1.52–1.53). Likewise, the odds of having SP antibodies increased by 33%, odds ratio of 1.33 (95% CI 1.22–1.45), and their level increased by 72%, rate ratio of 1.72 (95% CI 1.72–1.72), with unexplained variance at the level of participants of 3.32 (5391 observations among 1306 participants) and 1.18 (5390 observations among 1306 participants), respectively. The global antibody count predicted by the model is marked in red.

## Discussion

This study shows that in July/August 2020, 4 months after the first reported COVID-19 case in DRC, 17.3% of HCWs and 7.8% of their HH members in Kinshasa tested positive for the presence of SARS-CoV-2 antibodies, which increased to 62.1 and 31.2% by January 2022, respectively. The seroprevalence of the first survey found in our study lies within the range of what was reported in May and June 2020 among HCWs from Malawi [6] but is lower than that reported in HCWs from Bukavu, DRC, in July and August 2020 (41.2%) [8] and in Ibadan, Nigeria (45.1%) [7]. However, we have to be cautious when comparing seroprevalence results across different studies, as the figures can be influenced by the study design and the epidemiological context but also by the choice of the diagnostic tests, which tend to have differential sensitivity and specificity in the African setting [22, 23]. The high seroprevalence in the first survey of our study was somehow surprising, given the low number of clinical COVID-19 cases (10,401 PCR positives) and deaths (267) by September 15th, 2020, in Kinshasa, DRC. This discordance between serology and confirmed cases was subsequently observed in other sero-surveys in Africa. At the same time, in Belgian HCWs, for example, the seroprevalence was approximately 8% in a setting with 98,600 PCR positives (mainly clinical cases) and 10,000 COVID-19-related deaths [24]. Underreporting of mild clinical cases is a plausible reason within the Kinshasa setting, but it would not explain the low number of hospitalized cases or deaths, unless another factor is interfering. Possible explanations evidenced to date are a high proportion of the young population often associated with asymptomatic infections and the reduced risk of severe COVID-19 in African patients with parasite coinfection, such as helminth infection [25]. After the high seroprevalence at baseline, there was a decrease and subsequently an increase in seroprevalence, coinciding with reported symptomatic cases, as can be observed in the epidemiological curve of COVID-19 in DRC. It should also be noted that in the first months after pandemic declaration, some containment measures were taken by the DRC government in the general population, and the provision of protective equipment was provided to HCWs, together with training on compliance with IPC measures.

All sociodemographic and professional categories were equally positive for COVID-19 antibodies, and there was also no association with self-reported exposure to known COVID-19 cases or with risk behavior. A few HCWs had taken a PCR COVID-19 test in the first months of the pandemic, but neither this nor the presence of typical COVID-19 symptoms was associated with seropositivity. The finding of a higher seropositivity in the Bandalungwa health zone for the HCWs cannot be explained by the routine epidemiological surveillance data, which did not detect a cluster in this zone. Spatial clusters were not reported in the serological survey conducted in the general population of Kinshasa after the first wave of the COVID-19 pandemic [14]. The increasing seroprevalence over time, together with the absence of association with work-related or general risk behaviors and with HCW positivity in subsequent rounds in HH members, shows the importance of the time-dependent force of infection in a context where control measures were poorly followed by the population.

The IgG seroreversion rate was high between the first and second surveys (September 2020, 6 months after the first detected case) and was similar to the cohort of mild and asymptomatic COVID-19 cases followed up in North Carolina, USA, with 75.6% seroreversion over 5 months [26], but higher than the 39.5% seroreversion rate over 5 months in Connecticut, USA, in a cohort of mixed symptomatic and asymptomatic cases [27]. In our cohort, only 5 HCWs remained positive over the entire study period, which is much lower than that observed in other studies [28, 29], where the setting and proportion of symptomatic cases were different. Several factors may explain the seroreversion observed in our study. First, it has been shown that the magnitude of the immune response as well as the longevity of the antibodies are lower among asymptomatic infections [11, 30], which was the case for almost the totality of infections described here. In addition, even though the Luminex assay we used detected IgG, which was reported to last longer than IgM [31], we considered as positive only samples with concomitant presence of anti-spike and anti-nucleocapsid antibodies. Hence, any drop of one of the two antibodies was considered a seroreversion. In the UK, it was observed in primary school children and staff that anti-nucleocapsid antibodies stayed positive longer than anti-spike antibodies [32]. The patterns of seroprevalence over time fluctuated, with a decrease in seroprevalence at the second and fourth visits. A similar downward trend has also been found in other studies [10, 33–35]. These fluctuations have to be seen within the context of the COVID-19 epidemic in Kinshasa and the balance between seroreversion and seroconversion (Fig. 4). We showed that SARS-CoV-2 IgG antibody levels are dynamic over time in this African setting with low clinical case rates. This has implications for epidemiological studies; for example, if IgG levels fall below detection thresholds before they are measured, past infections may be underascertained, and spread of the virus could even be higher than observed in our study.

The WHO recently published a document [15]‘Toolkit for Integrated Serosurveillance of Communicable Diseases in the Americas’, where the potential uses of sero-surveys are discussed. On the basis of this document, we revisited the usefulness of sero-surveys within the COVID-19 pandemic in the setting of DRC, where relatively few clinical cases were described up to the end of 2021 and with a very low vaccination coverage of 21% in mid-2023 [12].

Potential uses of a sero-survey:

(1) – Estimate burden of disease. This was indeed one of the main objectives in the DRC study, as there was a very low availability of PCR testing for acute disease in the first months after the start of the pandemic, and there were very low numbers of symptomatic COVID-19 cases. The difficulty lies in the choice of a serological test. Within the first sero-survey round, different tests were used, and the congruence between test results was low, as described by Nkuba et al. [36]. In such conditions of a new disease, with uncertainty about case presentation and uncertainty about test interpretation, communication of findings to policy makers is hampered and does not aid the development of disease control strategies. During the subsequent rounds, using the same tests, interpretation of the trend was possible and gave, in addition to the reported COVID-19 cases, insight into the extent of transmission over time. It was evidenced that virus circulation was more important than based on clinical case reports.

(2) – Estimate the size of the population susceptible to disease and inherent risk for outbreaks and monitor changes in immunity over time due to exposure, infection, or interventions. At the start of the COVID-19 pandemic, it was not known that seroreversion would happen so quickly. We discovered seroreversion in the second survey in September 2020 but doubted these results, as the serological test was new and we had incongruent findings among the different tests used in the first survey [37]. This made us hesitant to report on seroreversion and hence the quick lowering of immunity, which is an important finding for policy makers, as it means that transmission is hardly lowered due to immunity after infection. We could follow up seroconversion and seroreversion in a setting with very low vaccination coverage up to the beginning of 2022.

(3) – Characterize patterns of pathogen transmission, monitor changes in pathogen transmission and investigate causes of the resurgence of diseases. We could indeed evaluate the link of transmission between HCWs and their households. Our main research hypothesis was that HCWs are bringing virus to their homes, and indeed, HCWs had higher seropositivity than HH members, but in the subanalysis, it was seen that household members did not have a higher risk of infection in the round after an HCW was positive. This result could only be obtained by the end of the study after several rounds, when there were enough observations. Hence, our initial hypothesis was rejected, but our results came too late to inform policy makers.

(4) – Identify high-risk groups. We could indeed identify risk groups, such as females, those who were older and those who were HCWs. However, these are not modifiable risk factors, and no intervention could be identified, except the priority of health care workers for vaccination campaigns, which was an obvious choice made in all countries affected by the pandemic. More important than identifying high risk for infection is determining who is at high risk of severe disease (and death). This has become clear quite early in the pandemic from clinical sites across the world: the elderly and those with comorbidities (especially diabetes, obesity, hypertension).

(5) – Determine the duration of immunity and detect the reintroduction or reemergence of diseases, monitor progress toward elimination goals and identify immunity gaps, establish theoretical herd immunity thresholds, and evaluate the impact of interventions. Due to the design of our study, sampling participants at fixed intervals, and the lack of availability of PCR tests, it was not possible to provide evidence on the duration of immunity. COVID-19 vaccines were hardly accepted in DRC, which started vaccinating on April 19th of 2021 [12], and with only 45 participants reporting being vaccinated between the fifth and sixth rounds, we could not evaluate the effect of interventions on the transmission force.

(6) – Summarizing evidence to provide a strong rationale and useful information that can be used to set priorities and guide policies and strategies for disease control and elimination. During the study, we were disappointed that it was not possible to have a direct impact on policy making. As it was a new disease and at time of the study no information on duration of antibodies was available, there was the doubt on the interpretation of serological results in general and of the sero-reversion in the second survey. This could have been important for policy, as this indicates the absence of increasing immunity or protection based on a natural infection. In subsequent surveys, we could follow up on the trend of transmission, which was higher than expected based on the reported cases, and the importance of seroreversion and seroconversion, but this did not provide much evidence for policy makers.

Although sero-surveys avoid the limitation of passive disease reporting systems, which can be unreliable due to underdiagnosis and undernotification, it turned out that the seroprevalence surveys were less useful than we had hoped for before the start of the study. This was due to the inherent characteristics of the disease and was not dependent on the design or rigor of the study. The evidence provided probably provides more information on what needs to be taken into account when performing sero-surveys in future outbreaks. The sero-survey was useful in the earliest days to realize that transmission of SARS-CoV-2 was very widespread, largely pauci- or asymptomatic. Afterwards, the information became less relevant, as infection and reinfection rates continued to be high, with seropositivity being a very poor predictor of protection against infection.

## Conclusion

Epidemic dynamics result from an interaction between the spread of infection, built immunity, demographic migration and waning immunity [38]. Understanding this interaction is key, and serological surveys can provide information on this immunity landscape for many infectious diseases, yet this methodology remains underexploited and interpretation hampered in a situation of a new disease with an unclear serological profile and unclear clinical case presentation.

## Data Availability

No datasets were generated or analysed during the current study.

## Declarations

**Ethical statement**.

The current study was approved by the ethics committee of the University of Antwerp, Belgium (number B3002020000144) and the national DRC ethics committee (189/CNES/BN/PMMF/2020). Further approval was obtained from the Ministry of Health through the provincial health division as well as the heads of concerned health zones (HZ) and health facilities. Prior to inclusion, the purpose of the study was explained to each potential participant, and written informed consent was obtained.
